# The Effect of Out of Hours Presentation with Acute Stroke on Processes of Care and Outcomes: Analysis of Data from the Stroke Improvement National Audit Programme (SINAP)

**DOI:** 10.1371/journal.pone.0087946

**Published:** 2014-02-12

**Authors:** James T. P. Campbell, Benjamin D. Bray, Alex M. Hoffman, Sara J. Kavanagh, Anthony G. Rudd, Pippa J. Tyrrell, on behalf of the Intercollegiate Stroke Working Party

**Affiliations:** 1 Royal College of Physicians, London, United Kingdom; 2 King's College London, Division of Health and Social Care Research, London, United Kingdom; 3 National Institute for Health Research Comprehensive Biomedical Research Centre, Guy's and St Thomas' NHS Foundation Trust, London, United Kingdom; 4 University of Manchester MAHSC, Salford Royal NHS Foundation Trust, Salford, United Kingdom; Imperial College London, Chelsea & Westminster Hospital, United Kingdom

## Abstract

**Background:**

There is inconsistent evidence that patients with stroke admitted to hospital out of regular working hours (such as weekends) experience worse outcomes. We aimed to identify if inequalities in the quality of care and mortality exist in contemporary stroke care in England.

**Methods:**

SINAP is a prospective database of acute stroke patients, documenting details of processes of care over the first 72 hours. We compared quality of care indicators and mortality at 72 hours, 7 days and 30 days, for patients who arrived within normal hours (Monday–Friday 8am to 6pm) and for those who arrived out of hours, using multivariable logistic and Cox proportional hazard models. Quality of care was defined according to time from arrival at hospital to interventions (e.g., brain scan), and whether the patient received therapeutic interventions (such as thrombolysis).

**Results:**

45,726 stroke patients were admitted to 130 hospitals in England between 1 April 2010 and 31 January 2012. Patients admitted out of hours (n = 23779) had more features indicative of worse prognosis (haemorrhagic stroke, reduced consciousness, pre stroke dependency). Out of hours admission was significantly associated with longer delays in receiving a CT scan or being admitted to a stroke unit, and reduced odds of receiving thrombolysis. After adjusting for casemix, there was no consistent evidence of higher mortality for patients admitted out of hours, but patients admitted at the weekends had a higher risk of 30 day mortality (OR 1.14, 95% CI 1.06–1.21)

**Conclusion:**

Inequalities in the provision of stroke care for people admitted out of regular hours persist in contemporary stroke in England. The association with mortality is small and largely attributable to higher illness severity in patients admitted out of hours.

## Introduction

Stroke is the third commonest cause of death [1] and the commonest cause of complex disability [2] in the UK. There is evidence that some interventions (for example stroke unit admission [3] and thrombolysis for acute ischaemic stroke [4]) improve outcome. “Out of hours care”, particularly weekend care, is associated with reduced staffing levels, fewer senior staff, more cross cover, and reduced access to imaging and other services [5], [6]. Across a number of care settings, including emergency admissions [7], [8], [9] pulmonary embolism [10] and myocardial infarction [11], admission at nights and weekends is reported to be associated with worse outcome. Specifically in stroke, there have been a number of studies that suggest that weekend admission is associated with higher in-hospital mortality [12], [13]. Admission at weekends with intracerebral haemorrhage, one subtype of stroke, is associated with increased risk adjusted in hospital mortality [14]. There is evidence from a previous cohort in the UK that weekend admissions with acute stroke are less likely to be admitted to a stroke unit, receive thrombolysis or have a CT scan than those admitted during the week [15]. There have been major changes in the organisation of stroke care since this study was published and it is not known if these changes have reduced inequalities in care for patients admitted out of regular working hours.

We aimed to review data from a large prospective dataset of acute stroke admissions in England to compare the quality of care and mortality in patients admitted out of hours (weekday evenings and overnight, weekends and public holidays) to determine whether inequalities in stroke care for patients admitted out of hours persist.

## Methods

The Stroke Improvement National Audit Programme (SINAP) is a continuous, prospective national clinical audit of acute stroke care in England, coordinated by the Royal College of Physicians London. The aim of SINAP is to audit against national guidelines on best practice for stroke patients in order to inform hospitals about their current performance and to identify national performance against key measures. 130 out of 186 (70%) hospitals in England which admit patients with acute stroke submitted data to the audit. Hospitals are encouraged to enter data on all admissions to the hospital with stroke regardless of ward to which they were admitted or what treatment they received. Patients with subarachnoid haemorrhage are not included. Uniformity in approach to data collection is achieved by providing audit participants with help notes on how to interpret the questions in the dataset and a dedicated email and telephone helpdesk to clarify queries about the dataset. The audit documents aspects of process of care in the first 72 hours of admission, including time from arrival to assessment by the stroke team, to CT scan and to a stroke unit, and interventions such as thrombolysis and administration of anti-platelet agents. Data were submitted by hospitals via a secure web tool with built-in validation checks. Mortality is reported via data linkage with the national death register (the Office for National Statistics).

Patients admitted between 1 April 2010 and 31 January 2012 are included in the cohort. Admission data including time of onset (where known), arrival at hospital time, stroke team assessment time, brain scan time and time of admission to the stroke unit are recorded. The dataset includes patient characteristics (including stroke type and severity) and interventions (such as thrombolysis or admission to stroke unit). There are also 5 care “bundles” which demonstrate the achievement of multiple related care processes on an “all or none” basis. These bundles were defined by the Intercollegiate Stroke Working Party. Patients can be excluded from care bundles based on pre-specified exclusion criteria. ‘Normal hours’ are defined as weekdays between 8am and 6pm and ‘out of hours’ as weekdays before 8am or after 6pm or at any time on a weekend day or English public holiday. Patients who were already in hospital at time of stroke (2,871 patients, 5.9%) are excluded from the analysis. Patients defined in the audit as eligible for thrombolysis have the following characteristics: ischaemic stroke; age less than 81 years; an onset of symptoms to arrival at hospital time of less than 3 hours; no contra-indications for thrombolysis; and did not decline treatment. These eligibility criteria are based on European licensing indications at the time.

The aim of our study was to determine whether patients admitted out of hours to hospital with acute stroke have worse care or higher mortality than people admitted within hours. Logistic regression was used to test the relationship between arrival category and outcomes. Mortality was analysed at 72 hours, 7 days and 30 days after admission. Sensitivity analyses were carried out in which patients dying within the first 3 days following admission were excluded, on the basis that such early mortality may be a marker of stroke severity and co-morbidities, rather than reflect process of care. A further sensitivity analysis was carried out adjusting for stroke syndrome type using the Oxford classification [16] (OCSP). A Cox proportional hazards model was used to test the relationship between arrival category and time taken for patients to progress along the stroke care pathway. In this analysis, a hazard ratio above one is interpreted as faster progress, and below one as slower progress. Analyses were adjusted for age, sex, worst level of consciousness in the first 24 hours (a surrogate for stroke severity), stroke type (ischaemic versus primary intracerebral haemorrhage) and pre stroke independence. Regression analysis results are presented as odds ratios or hazard ratios with analytical 95% confidence intervals. Categorical data are presented as N (%), and continuous data are presented as mean (SD) or median (IQR) as appropriate. All analysis was performed using Stata 12.1.

### Ethics Statement

Ethical approval of the SINAP audit and associated data linkage was granted by the Ethics and Confidentiality Committee of the National Information Governance Board. This included Section 251 (under the NHS Act 2006) approval to collect data without active patient consent and patients are able to request for any identifiable data to not be included. Patients and patient representatives are involved in the design, reporting and oversight of SINAP.

## Results

Of the 45,726 stroke patients, 21,947 patients (48%) arrived at hospital within normal hours and 23,779 patients (52%) arrived out of hours. Whilst the two groups were similar in terms of age and sex, there were significant clinical differences between them [Table 1]. There were a greater proportion of patients with primary intracerebral haemorrhage amongst the out of hours group (12.2% versus 10.0%, p<0.0001), fewer patients with lacunar infarct (16.6% versus 18.0%, p<0.001) and fewer patients who were fully conscious in the first 24 hours (73.8% versus 78.2%, p = 0.003). In univariable analysis, out of hours patients had a significantly higher mortality at 72 hours (2.3% versus 1.9%, p = 0.007), 7 days (6.5% versus 5.8%, p<0.001) and 30 days (14.3% versus 12.1%, p<0.001).

**Table 1:** Demographics and unadjusted mortality for normal hours versus out of hours patients.

	Normal hours	Out of hours	All	p value
n (%) (unless otherwise specified)	N = 21947	N = 23779	N = 45726	
Records per site, median (IQR)*	122 (39, 242)	125 (41, 253)	250 (77, 497)	
Sex: Female	11213 (51.1)	12070 (50.8)	23283 (50.9)	
Male	10734 (48.9)	11709 (49.2)	22443 (49.1)	0.478
Age, median (IQR)*	77 (66, 84)	77 (66, 84)	77 (66, 84)	0.067
Males age, median (IQR)*	73 (63, 81)	73 (63, 81)	73 (63, 81)	0.484
Females age, median (IQR)*	80 (71, 87)	80 (71, 87)	80 (71, 87)	0.137
Aged 81 or over	8347 (38.0)	8982 (37.8)	17329 (37.9)	0.567
Independent in everyday activities pre-stroke	17479 (79.6)	18639 (78.4)	36118 (79.0)	0.003
Type of stroke: Infarction	19686 (90.0)	20775 (87.8)	40461 (88.9)	
Primary Intracerebral Haemorrhage	2185 (10.0)	2883 (12.2)	5068 (11.1)	<0.001
OCSP** Stroke classification (Infarct only) TACI	2324 (11.8)	2564 (12.3)	4888 (12.1)	
LACI	3535 (18.0)	3445 (16.6)	6980 (17.3)	
POCI	1873 (9.5)	1848 (8.9)	3721 (9.2)	
PACI	11391 (57.9)	12315 (59.3)	23706 (58.6)	
Other	563 (2.9)	603 (2.9)	1166 (2.9)	<0.001
Worst consciousness level in first 24 hours: Fully conscious	17154 (78.2)	17536 (73.8)	34690 (75.9)	
Drowsy	3124 (14.2)	4012 (16.9)	7136 (15.6)	
Semi-conscious	866 (4.0)	1113 (4.7)	1979 (4.3)	
Unconscious	803 (3.7)	1118 (4.7)	1921 (4.2)	<0.001
Palliative care decision in first 72 hours	1178 (5.4)	1654 (7.0)	2832 (6.2)	<0.001
72 hour mortality	426 (1.9)	548 (2.3)	974 (2.1)	0.007
7 day mortality	1262 (5.8)	1675 (7.2)	2937 (6.5)	<0.001
30 day mortality	2619 (12.1)	3337 (14.3)	5956 (13.2)	<0.001

* Inter quartile range.

** Oxford Community Stroke Project.

**Figure 1:**
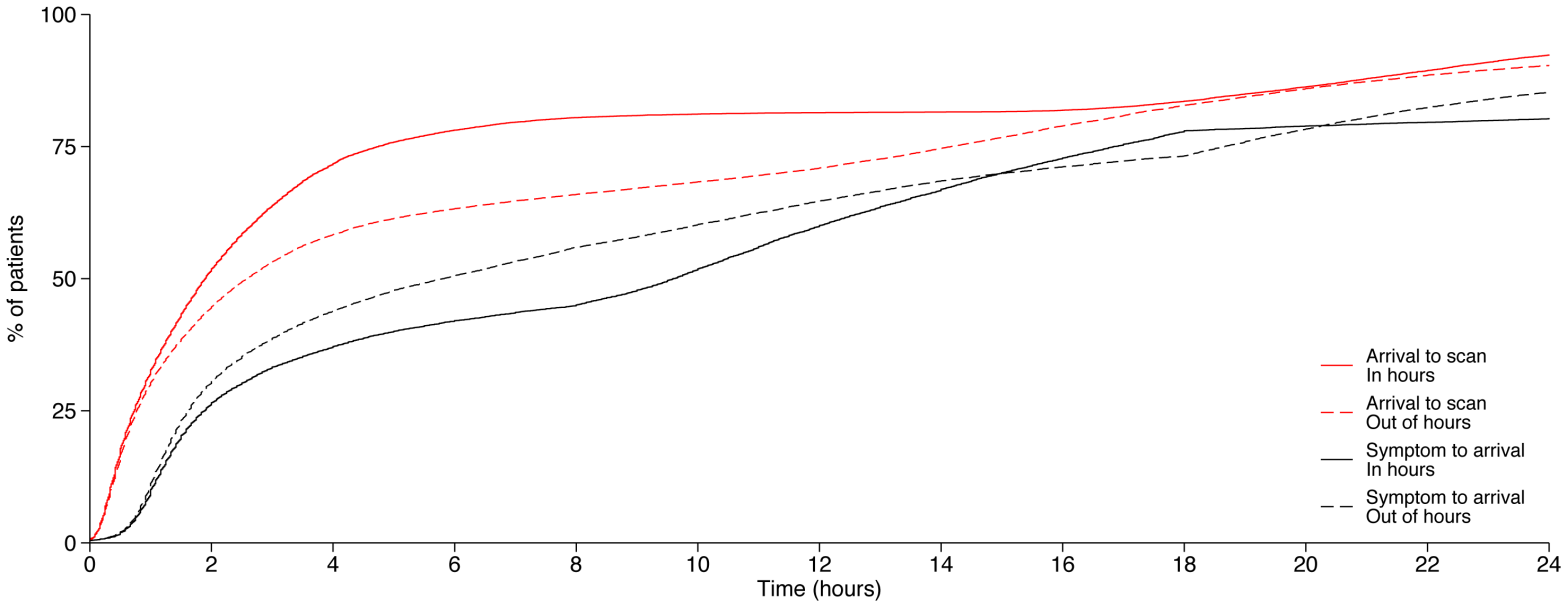
Cumulative frequency plot of arrival to scan and symptom to arrival delays for normal hours and out of hours patients.

In univariable analysis, there was a significant difference (favouring normal hours arrivals) in the proportion of patients receiving several audited processes of care (Table 2). A higher proportion of patients arriving in normal hours received Bundles 1, 3 and 5 and thrombolysis if eligible, and a lower proportion were catheterised. These patients also had faster arrival to scan (Figure 1; median 114 minutes *versus* 155 minutes, p<0.001) and arrival to stroke unit times. There was no significant difference between the two arrival groups for Bundles 2 and 4. A greater proportion of out of hours patients were estimated to be eligible for thrombolysis, based on shorter times from onset to admission (median 349 *versus* 576 minutes, p<0.001) (Table 2 and Figure 1).

**Table 2:** Unadjusted results for processes of care comparing normal hours with out of hours patients.

	Normal hours	Out of hours	All	p value
n (%) (unless otherwise specified)	N = 21947	N = 23779	N = 45726	
Bundle 1	10315 (54.4)	9255 (47.0)	19570 (50.7)	<0.001
Bundle 2	16362 (84.2)	17695 (84.3)	34057 (84.3)	0.484
Bundle 3	12259 (55.9)	11921 (50.1)	24180 (52.9)	<0.001
Bundle 4	11130 (59.5)	11860 (58.7)	23190 (59.1)	0.122
Bundle 5	7451 (41.2)	6845 (35.4)	14296 (38.2)	<0.001
Eligible for thrombolysis	2002 (9.1)	2606 (11.0)	4608 (10.1)	<0.001
Thrombolysed if eligible	1252 (62.5)	1350 (51.8)	2602 (56.5)	<0.001
Given oxygen if required	3578 (68.5)	4379 (72.1)	7957 (70.5)	<0.001
Catheterised (except for retention)	1507 (6.9)	1972 (8.3)	3479 (7.6)	<0.001
Onset to arrival time, median (IQR) in minutes	576 (113, 1010)	349 (97, 1120)	462 (103, 1063)	<0.001
Arrival to stroke unit time, median (IQR) in minutes	209 (120, 383)	227 (127, 741)	218 (123, 505)	<0.001
Arrival to scan time, median (IQR) in minutes	114 (44, 285)	155 (46, 850)	130 (45, 644)	<0.001

•Bundle 1: The patient was seen by a nurse (trained in stroke management) and one therapist within 24 hours of hospital arrival and all relevant therapists within 72 hours.

•Bundle 2: The patient had a nutrition screening and, when required, a formal swallow assessment within 72 hours.

•Bundle 3: The patient's first ward of admission was a stroke unit and they arrived there within four hours of hospital arrival.

•Bundle 4: The patient was given an antiplatelet, when appropriate, within 72 h and adequate fluid and nutrition in all 24 hour periods.

•Bundle 5 includes the following stroke standards: Admitted to stroke unit within 4 hours; Scanned within 24 hours; Seen by stroke consultant or associate specialist within 24 hours; Saw nurse within 24 hours; Nutrition screening within 72 hours; Prognosis/diagnosis discussed with relatives/carers within 72 hours; Physiotherapy assessment within 72 hours.

**Figure 2:**
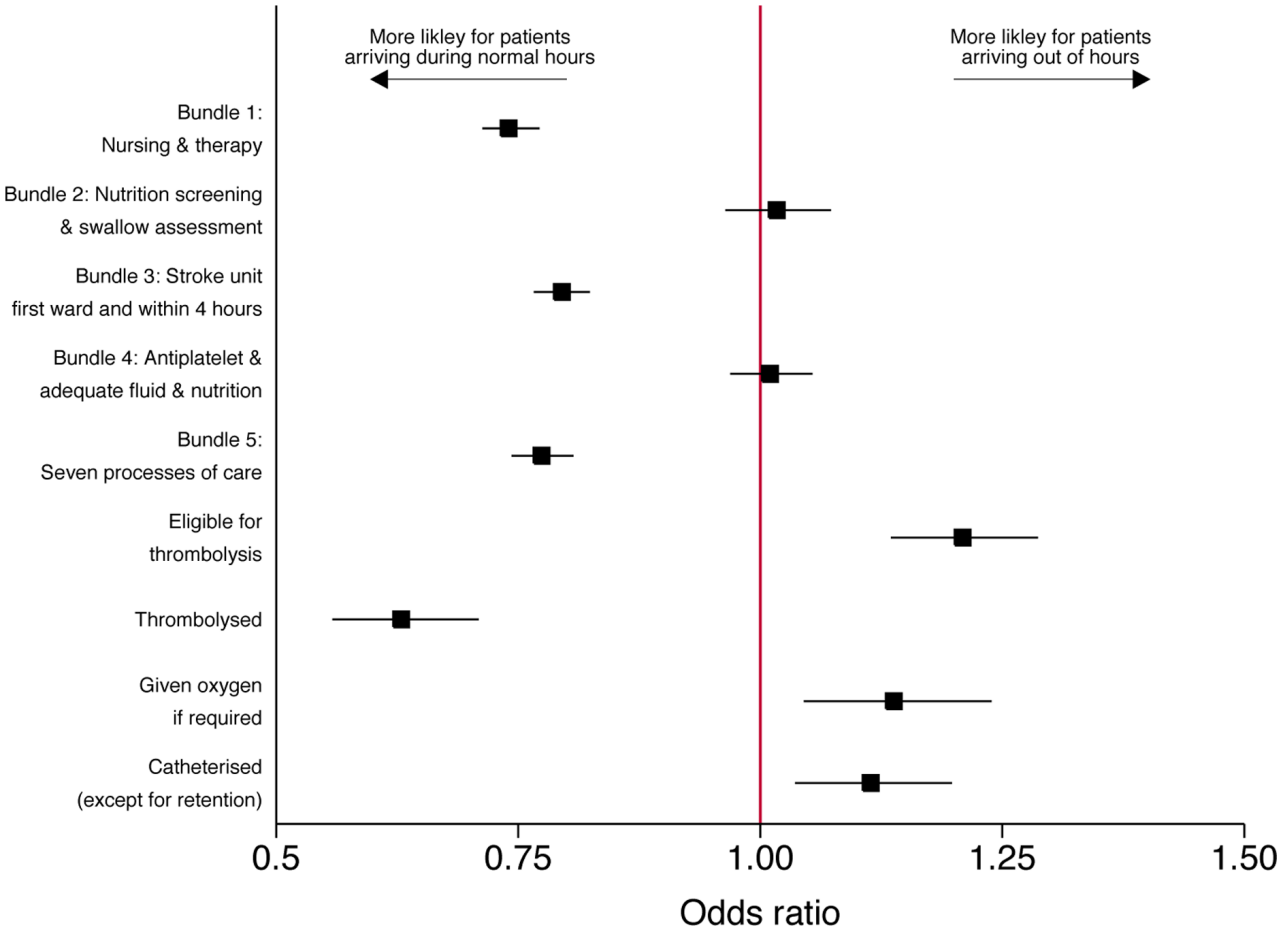
Eligibility for and compliance with process measures for normal hours and out of hours patients (adjusted odds ratios).

**Figure 3:**
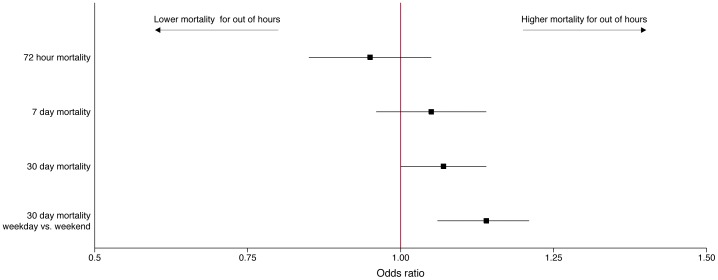
Adjusted mortality odds ratios for normal hours and out of hours patients.

Similar associations were observed in the multivariable analysis of process of care [Table 3 and Figure 2]. Out of hours patients had a higher odds being eligible for thrombolysis (OR 1.25, 95% CI 1.17–1.33, p<0.001), but reduced odds of receiving it if eligible (OR 0.62, 95% CI 0.55–0.70, p<0.001). The adjusted mortality results are shown in Figure 3 and Table 4. After adjusting for patient casemix, there were no significant differences in 72 hour and 7 day mortality for out of hours admissions. There was a small higher risk of 30 day mortality, but only at a borderline level of statistical significance (OR 1.07, 95% CI 1.00–1.14, p = 0.040). Using a different measure of out of hours (weekends but not overnight) yielded a slightly higher odds ratio of death (OR 1.14, 95%CI 1.06–1.21, p<0.001). In sensitivity analysis, excluding the 72 hour deaths increased the odds ratio of 30 day mortality for out of hours patients, (OR 1.10, 95% CI 1.03–1.18, p = 0.008). Patients admitted out of hours were not at higher risk of mortality when adjusting for OCSP subtype (OR 0.96, 95% CI 0.86–1.06, p = 0.41).

**Table 3:** Adjusted results for processes of care comparing normal hours with out of hours patients.

		Univariable		Multivariable*	
	N of patients	OR	p	OR	p
Bundle 1 (nursing and therapy)	38504	0.74 (0.71–0.77)	<0.001	0.74 (0.71–0.77)	<0.001
Bundle 2 (nutrition screen and swallow assessment)	40270	1.02 (0.97–1.08)	0.48	1.02 (0.97–1.08)	0.44
Bundle 3 (stroke unit direct admission)	45529	0.79 (0.77–0.82)	<0.001	0.80 (0.77–0.83)	<0.001
Bundle 4 (fluid/nutrition/antiplatelet)	39113	0.97 (0.93–1.01)	0.12	1.02 (0.98–1.07)	0.32
Bundle 5	37292	0.78 (0.75–0.81)	<0.001	0.78 (1.75–0.81)	<0.001
Eligible for thrombolysis	40461	1.27 (1.19–1.35)	<0.001	1.25 (1.17–1.33)	<0.001
Thrombolysed if eligible	4608	0.64 (0.57–0.73)	<0.001	0.62 (0.55–0.70)	<0.001
Given oxygen if required	11236	1.19 (1.09–1.29)	<0.001	1.13 (1.03–1.23)	0.007
Catheterised (except for retention)	45529	1.23 (1.14–1.32)	<0.001	1.10 (1.02–1.18)	0.011

	N	HR	p	HR	p
Onset to arrival time	40138	1.12 (1.10–1.14)	<0.001	1.10 (1.08–1.13)	<0.001
Arrival to stroke unit time	42601	0.86 (0.85–0.88)	<0.001	0.87 (0.85–0.89)	<0.001
Arrival to scan time	42601	0.85 (0.84–0.87)	<0.001	0.84 (0.85–0.89)	<0.001

* Adjusted for age, sex, worst level of consciousness in the first 24 hours, stroke type and pre-stroke independence.

Odds and hazard ratios for achieving each of the process measures. Reference category is normal hours patients.

**Table 4:** Adjusted mortality results.

		Univariable		Multivariable*	
	N of patients	OR	p	OR	p
72 hour mortality	44783	1.17 (1.06–1.29)	0.002	0.95 (0.85–1.05)	0.32
7 day mortality	44783	1.24 (1.15–1.34)	<0.001	1.05 (0.96–1.14)	0.28
30 day mortality	44783	1.21 (1.14–1.27)	<0.001	1.07 (1.00–1.14)	0.040
30 day mortality comparing weekend admission with weekday admission	44783	1.24 (1.16–1.32)	<0.001	1.14 (1.06–1.21)	<0.001

* Adjusted for age, sex, worst level of consciousness in the first 24 hours, stroke type and pre-stroke independence.

Reference category is normal hours patients, except for last row which is patients admitted any time on a weekday.

## Discussion

This study shows that there are significant differences both in the patient population admitted with stroke out of usual working hours, and in the quality of the care that they receive. Patients admitted out of hours are more likely to present with haemorrhagic stroke, have reduced consciousness and have pre-morbid dependency; those patients with ischaemic stroke are more likely to present with more severe stroke subtypes. These data suggest that the observed excess mortality associated with out of hours admission reported in previous studies can largely be explained by un-measured differences in severity and prognosis. However, despite presenting with a greater illness severity, patients admitted out of hours are also less likely to receive timely access to key investigations and interventions, such as brain scanning and stroke unit admission. These data show that in this large sample of contemporary stroke care in England, there remain significant inequalities in care standards depending on the time of day and day of week that a patient has a stroke.

### Comparison with Other Studies

Several previous observational studies have demonstrated excess mortality associated with out of hours or weekend admission for acute stroke, although the effect size has generally been modest and not all studies have demonstrated such an effect. In the largest previous cohort study from England, patients admitted on a Sunday were found to have 26% higher odds of inpatient mortality compared to those admitted on a weekday [17]. However, the study was based on administrative data returns and was therefore limited in its ability to control for stroke severity between weekend and weekday admissions. Data from the Canadian Stroke Registry showed a similar excess risk of 7 day mortality in patients admitted at weekends [18]. More modest estimates of excess mortality risk were observed in data from the US Get With The Guidelines register, where out-of-hours admission was associated with 9% and 19% higher odds of in-patient mortality in patients with ischaemic stroke and haemorrhagic stroke respectively [13]. By contrast, studies in other settings have not observed higher mortality in weekend admissions [19], [20]. In our study, no significant differences were observed in mortality at 72 hours or 7 days for patients admitted out of hours when adjusting for patient casemix. 30 day mortality was higher in some but not all of the analyses, and with only borderline levels of statistical significance. These suggest that more complete adjustment for casemix can explain most if not all of the observed excess mortality for patients admitted out of hours. Patients admitted out of hours had a greater number of features associated with worse prognosis (such as haemorrhagic stroke, reduced consciousness, dependency in activities of daily living prior to stroke). These differences in casemix may reflect differences in access to community medical care services out of hours, less awareness of minor stroke symptoms that occur during sleep or reduced propensity of patients to seek medical attention out of regular hours. Interestingly, patients admitted out of hours presented to hospital quicker after the onset of their symptoms. This may reflect greater urgency in referral and transfer to hospital in response to more severe acute symptoms (such as reduced consciousness) or possibly may reflect differences in pre-hospital transport (reduced demand for ambulances, lower traffic levels) for out of hours admissions.

There was a stronger association with weekend admission and mortality, which was associated with a 14% higher odds of 30 day mortality. The reasons for differences between weekend admission compared to a broader definition of out of hours care (including overnight admissions) are not clear. It is possible that the effect of weekend admission on mortality relates to the prolonged period of exposure to out of hours working (up to 56 hours versus up to 14 hours for overnight admissions).

This study found significant differences in the quality of care received by patients admitted out of hours. Patients admitted out of hours waited longer to receive a brain scan or be admitted to a stroke unit and were less likely to be admitted to a stroke unit directly or to receive thrombolysis, multidisciplinary stroke specific care and therapy early after admission (Bundle 1). Other aspects of care quality were equivalent, including nutritional and swallow screening and administration of antiplatelet therapy, adequate fluid and nutrition. Differences in care quality may reflect the differences in the organisation of out of hours care – access to brain scanning may be restricted out of hours, and many hospitals have reduced nursing and medical staff rostered at weekends [16]. In particular, the difference in thrombolysis rates suggests that patients admitted out of hours have inequitable access to one of the key evidence based therapies for acute stroke [4]. This is even more unfortunate given that patients presenting out of hours presented quicker to hospital and thus potentially have most to gain from timely administration of thrombolysis [21].

These findings are similar to those reported in the United Kingdom from 2005, which demonstrated reduced access to early CT scanning and stroke unit admission for weekend admissions. Since then, changes in stroke service organisation in England have significantly increased the number of stroke unit beds and CT scan availability (RCP Sentinel Audit 2010). These data show however that these improvements have not eradicated inequality in stroke care arising from out of hours admission. There is evidence that reorganisation for stroke care can reduce these inequalities – outcomes from out-of-hours admissions to Comprehensive Stroke Centres in the USA has been reported as being no worse for weekend admissions [20].

### Strengths and limitations

These data represent a large, national cohort of patients, with high levels of data completeness and linkage with national mortality records to generate accurate estimates of mortality rates. Several previous studies have used in-patient mortality as the outcome of interest: this may underestimate true mortality rates, particularly in a changing health care environment where patients may be discharged early to nursing home or intermediate care facilities. The dataset is also specifically designed to capture accurate information concerning the process of acute stroke care, and therefore is likely to give more accurate estimates than those derived from routine coding of administrative data. The outcomes data are however limited to mortality, and no data was available on other important stroke outcomes such as disability and quality of life. It is therefore not possible to determine if inequalities in process of care for patients admitted out of hours influenced these outcomes. Adjustment for stroke severity also was limited by lack of availability of the National Institutes of Health Stroke Scale (NIHSS), which is commonly used in stroke research, and other prognostic variables such as cardiovascular comorbidities. Adjustment for stroke severity was therefore carried out using other measures of severity (consciousness level and OCSP subtype). SINAP is not a population based register, and hospital participation is voluntary. Differences in case ascertainment and reporting between hospitals cannot therefore be excluded. Both the voluntary participation and differing case ascertainment may be a source of bias as non-participating hospitals may have different processes of care. Nevertheless, our results are based on patient level data rather than hospital level, so the effect of low and non-participation should not be overstated.

### Summary

Despite improvements in the organisation of stroke care in England over recent years, significant inequalities in care remain for patients with acute stroke admitted out of regular working hours. Observed excess mortality for patients admitted with stroke out of hours are largely explained by higher illness severity, although patients admitted at the weekend had a small but significant increased risk of mortality. Despite worse illness severity, patients admitted out of hours wait longer for key investigations and interventions, and are less likely to receive several aspects of multidisciplinary stroke care. Strategies to improve 24/7 emergency care generally should allow more hospitals to provide high quality specialist care regardless of time of presentation.
